# Updates on Nutraceutical Sleep Therapeutics and Investigational Research

**DOI:** 10.1155/2015/105256

**Published:** 2015-07-21

**Authors:** Michael Yurcheshen, Martin Seehuus, Wilfred Pigeon

**Affiliations:** ^1^University of Rochester School of Medicine and Dentistry, 601 Elmwood Avenue, Rochester, NY 14642, USA; ^2^Middlebury College, 14 Old Chapel Road, Burlington, VT 05753, USA; ^3^Veterans Administration, 400 Fort Hill Avenue, Canandaigua, NY 14424, USA

## Abstract

Approximately 50% of the population will suffer from a sleep disorder over the course of their lifetime. There is increasing interest in nutraceuticals for these conditions. The quality of the evidence for the safety and effectiveness of using these supplements to treat sleep disorders varies substantially. In this review, we discuss the data about the effectiveness and safety of six commonly used plant-based sleep therapeutics: caffeine, chamomile, cherries, kava kava, L-tryptophan, marijuana, and valerian. We explore both historical uses of each substance and the current state of the literature.

## 1. Introduction

Overall, it is estimated that over 50% of the US population will suffer from a sleep disorder at some point in their life [1]. These conditions include disorders of sleep initiation and maintenance (i.e., insomnia), hypersomnolence, and others. As a therapeutic group, there is an abundance of interest in nutraceuticals for these disorders. Many naturally occurring products are manufactured and marketed for a variety of sleep symptoms.

Comprehensive research on these natural products for sleep-related indications is in its adolescence, and there is a spectrum of evidence supporting the efficacy of these agents. The agents range from caffeine, with significant evidence to support its potential in several sleep conditions, to marijuana, with virtually no evidence, and many in between.

Here, we focus on six commonly recognized plant-based nutraceuticals with hypothesized impact on sleep disorders: caffeine, chamomile, cherries, L-tryptophan, marijuana, and valerian. The exclusion of melatonin from this focus is based upon two factors. First, although melatonin may be obtained from a variety of plant sources or from chemical synthesis, formulations have traditionally been derived from animal sources. Second, a number of general and systematic reviews of melatonin are already published in the literature.

The inclusion of any of the agents in this review does not imply endorsement; the agents were selected on the basis of common use in the general population in conjunction with public interest in their research and development. This review will summarize both the historic use and state of the literature for these agents and provide an assessment of the field's understanding of their sleep/wake effects.

## 2. Caffeine

Caffeine is among the most widely consumed plant-based drugs worldwide. With the exception of cannabinoid extracts, it is the only agent discussed in this review that has FDA recognized formulations. As such, caffeine is both a nutraceutical and a pharmaceutical product. It is also singular in this discussion of plant-based sleep therapeutics, in that it is used for conditions of excessive sleepiness, rather than the disorders of sleep initiation and maintenance.

There is a wide array of sources for naturally occurring caffeine [2, 3]. A typical 8 oz cup of coffee brewed from roasted beans contains roughly 100 mg of caffeine. Teas range from being completely uncaffeinated/decaffeinated to over 100 mg, depending on the type and brewing method. Chocolate and chocolate products contain caffeine, about 10 mg for a 1 oz serving of milk chocolate. Some sources are less well known in the United States. These include guarana, a plant native to South America where it is formulated into food consumables and ingested for its natural stimulating effects [4]. Caffeine (usually obtained from decaffeination processes) can also be added to artificial sources such as soda and energy drinks.

Caffeine (1,3,7-trimethylpurine-2,6-dione) has a number of biochemical actions. Its principal action is adenosine antagonism, and there are a number of caffeine analogs with variable affinities for A1 and A2 receptors [2]. Adenosinergic pathways upregulate non-REM sleep, and it is the antagonistic effect at these receptors that primarily accounts for caffeine's stimulant properties. It also acts as a cyclic AMP phosphodiesterase inhibitor, perhaps contributing to its alertness and mood elevating effects [3]. Caffeine has a number of other proposed bioactive mechanisms, including acetylcholinesterase antagonism that may account for its memory-enhancing properties [5]. In addition to caffeine, coffee and tea contain a number of antioxidants, but it is unclear if these have any particular beneficial action from a sleep or circadian standpoint [6].

On a macrolevel, these actions all contribute to caffeine's stimulant effects. In a rigorous Cochrane meta-analysis, caffeine was found to be effective in controlling errors secondary to shift work and is perhaps as effective as other (potentially more expensive and difficult to obtain prescription) therapies for this purpose [7]. It can also be effective in treating sleepiness in other types of circadian rhythm disorders, such as jet lag [8]. Although several FDA approved stimulants have been studied for residual sleepiness in treated sleep apnea, caffeine has not been studied for this malady. At one time, caffeine was suggested as a therapy for central sleep apnea, but it is no longer routinely used for treatment of this condition.

Caffeine has been demonstrated to improve performance with a number of psychomotor tasks. This quality can be useful both individually and from a public safety standpoint. For instance, caffeine may help to reduce the likelihood of motor vehicle accidents in commercial drivers. In one case-control study, Australian researchers polled 530 commercial drivers who had been involved in a recent motor vehicle accident and compared them to 517 control drivers. [9]. After adjustment for confounding factors, drivers who had consumed caffeine (specifically for the purpose of staying awake) had a 63% reduced chance of a crash (odds ratio 0.37, 95% confidence interval 0.27 to 0.50). This study had a number of potential methodological flaws, including recall bias, but suggests that caffeine could reduce the likelihood of a commercial vehicle crash and therefore promote public safety when used in this manner.

The side effects of caffeine are well recognized. Recently, there has been significant interest in establishing the safety of energy drinks, many of which contain significant amounts of caffeine. Agitation, insomnia, and appetite suppression are some of the more common side effects. Palpitations can be common with excessive caffeine ingestion; however, its role in precipitating more serious arrhythmias is speculative [10]. More recently, an association between caffeine intake and hemorrhagic stroke was established in a population based study, although the study examined caffeine ingested in processed form and not from caffeinated beverages [11]. A link between fibrocystic breast diseases has been suggested, but some studies cast doubt on this relationship [12]. In general, most of the side effects of caffeine are minor and self-limited.

## 3. Chamomile

Chamomile compound is derived from several plants in the* Asteraceae* family, native to southern and Eastern Europe. Chamomile has been traditionally served as an infusion, but a number of formulations are available, including tablets, powders, and gelcaps. As with virtually all nutraceuticals, chamomile is considered a dietary supplement and is not FDA regulated. Chamomile has a reputation as a natural aid for insomnia and has been used for millennia in this capacity. In common use, the supplement is generally considered to be soporific, rather than hypnotic, and is easy to find in brick and mortar and online retail outlets throughout the United States.

Chamomile contains several compounds that might have therapeutic effects [13]. In laboratory studies, Apigenin demonstrates anxiolytic properties and appears to be a candidate for chamomile's effectiveness as soporific [14–16]. Apigenin is a flavonoid and acts as an antagonist at *α*1*β*1*γ*2S GABA_A_ receptors and at *ρ*1 GABA_C_ receptors. GABA modulation is a common mechanism for many hypnotics, although these putative receptors are not those modulated by benzodiazepines or benzodiazepine agonists [17]. Apigenin has also been recently studied for additional properties, including anti-inflammatory effects, although the relationship between these and possible sleep inducing effects is still speculative.

The literature is sparse in terms of controlled trials examining chamomile and its sleep inducing properties. In a recent study, 34 subjects were randomized to placebo or 270 mg of chamomile extract [18]. This dosage is much higher than could be prepared and ingested as a tea. Chamomile did not demonstrate a statistically significant benefit over placebo in the predetermined primary sleep endpoints, specifically, sleep diary metrics. Similarly, there was no statistically significant impact on daytime functioning. This is the* only* high quality clinical trial published to date regarding clinical efficacy of chamomile, and the authors highlight its limitations, including small sample size and challenges in developing effective dosing. All other clinical studies do not meet class 1A evidence and are generally limited to case reports and series. According to Clinicaltrials.gov, there are no other trials that are recruiting or recently completed examining the effect of chamomile on sleep [19].

Like the record on effectiveness, the published literature examining the tolerability of chamomile is generally limited. The only randomized, controlled trial of chamomile did not demonstrate any adverse effects, but the sample size was limited [18]. In other reports, chamomile was linked to contact dermatitis, but only while used topically [20, 21]. Although several population based studies have examined the impact of supplements on premature labor and gestational weight, the largest of these did not find any association between chamomile and these outcomes [22]. With limited literature, definitive conclusions about chamomile's side effects are lacking.

## 4. Cherries and Cherry Juice

Of many varieties of cherry, tart cherries in particular are purported to have a number of beneficial health effects with some evidence supporting decreases in oxidative stress levels of inflammatory markers and muscle damage following exercise while enhancing muscle recovery [23–28].

Montmorency tart cherries (*Prunus cerasus*) contain several phytonutrients including the phenolic acids with anthocyanins chief among them [29]. Anthocyanin levels in tart cherries exceed those found in sweet cherries and other fruits and a positive linear relationship between anthocyanin levels in cherries and oxidative stress protection in neuronal cells has been reported [30, 31]. Tart cherries also contain high levels of anti-inflammatory substances and melatonin [29, 31, 32]. This suggests two putative sleep-promoting pathways for tart cherries (and perhaps some sweet cherries as well). First, the presence of anti-inflammatory cytokines in tart cherries suggests one potential pathway to sleep enhancement, given that a number of inflammatory cytokines are intricately related to the modulation of sleep [33]. Second, the relatively high content of melatonin in some cherries may serve as a source of exogenous melatonin, a substance with proven sleep-regulating properties. Only very limited clinical research, however, has been conducted to assess the impact of cherries on sleep.

In separate studies, sweet and tart cherries were associated with very modest sleep improvements in already good sleepers. In a crossover study (*n* = 20) of montmorency tart cherry juice blend, nonsignificant reductions in sleep latency (approximately 5 minutes) and 1 minute in wake after sleep onset (WASO) were measured from sleep diary data [34]. In an uncontrolled study (*n* = 12), seven varieties of Jerte Valley sweet cherries (*Prunus avium*) were consumed as whole fruits over seven, separate 72-hour periods. Actigraphy measured improved sleep outcomes compared to baseline [35]. Notably, both studies measured urinary melatonin levels and found increases over basal levels following cherry supplementation (and compared to placebo in the crossover study). Together, these two studies suggest that tart cherry juice and whole sweet cherries each increase melatonin levels in healthy sleepers, making more plausible the possibility that melatonin supplementation may improve sleep in a sleep disordered sample.

Only one study to date has investigated the effect of cherries in a clinical sample. In a small crossover study (*n* = 15) a montmorency tart cherry juice was associated with statistically significant improvements in self-reported sleep among older adults with insomnia [36]. Compared to placebo, subjects drinking tart cherry juice for two weeks reported less severe insomnia and a reduction in time awake following sleep onset compared to placebo. The magnitude of between-group effects was small to moderate in favor of the tart cherry juice, far less robust than observed from established pharmaceuticals. Nonetheless, the observed effects of tart cherry juice do also compare favorably with the use of exogenous melatonin for insomnia [37].

The amount of melatonin intake from the two tart cherry studies (0.08 mg) is less than the lowest doses of exogenous melatonin (0.3 mg) found to have an impact on sleep [38]. Moreover, the relatively rapid elimination half-life of melatonin (<1 hour) cast some doubt on the improvements seen in wake after sleep onset. It is possible that other mechanisms or pathways to improved sleep underlie the positive signal that remains with respect to cherries and sleep [39]. At this stage, the questions of if and how cherries promote or improve sleep in clinical samples remain unanswered, as does the mechanism of action by which such improvements may occur.

## 5. Kava Kava

Kava kava (*Piper methysticum*) is a plant native to several ecosystems of the Western Pacific, including Hawaii. The sedative and anxiolytic effects of the root from the various substrains of this plant have been known for millennia. Peoples of French Polynesia, for instance, have been using preparations of the plant for a variety of maladies, including symptoms of anxiety and depression. Most raw kava kava is produced and harvested outside of Western nations and imported into European and North American markets for processing. Kavalactones appear to be the primary psychoactive constituents contained in the plant, of which several have been identified. The putative receptors of these compounds include GABA, serotonin, and dopamine, among others [40].

By reputation, kava has a host of therapeutic effects, including anxiolytic, euphoric, and sedative properties. Of these, the anxiolytic benefits of the nutraceutical are probably the best characterized. A 2003 Cochrane report, republished in 2010, analyzed a total of 12 randomized, controlled studies with a total of 700 participants [41]. Several additional studies were excluded from the analysis, as they did not meet inclusion criteria. After rigorous analysis, the authors concluded kava is superior to placebo in controlling anxiety, but the effect size seemed small due to small numbers of subjects included in the trials.

In animal models, one kavalactone, kavain, appeared to change sleep micro- and macroarchitecture compared to other sedatives [42]. An analogous trial has not been conducted in humans. In fact, almost no randomized, controlled trials have explored the efficacy of kava kava in treatment for insomnia and the few published trials have generally included sleep metrics as a secondary outcome measure. A poorly conducted trial published in 2004 examined the impact of a kava extract on sleep scores on a validated sleep questionnaire in subjects with baseline anxiety [43]. After excluding a number of potential subjects, the authors compared 34 subjects taking kava extract to 27 subjects taking placebo. They saw a statistically significant benefit to kava compared to baseline in their predetermined metrics, but both groups saw significant improvements. In the absence of high quality, randomized controlled trials, there is clear opportunity to explore the efficacy of kava in primary insomnia.

Kava has been subject to a number of safety concerns, and this itself is a matter of controversy [44]. The primary one amongst these is the possibility of hepatotoxicity. Due to concerns about this specific side effect as well as other regulatory matters, kava has been highly restricted, particularly in the European Union where it had been banned from import for a number of years. Several authors have suggested that the mode of preparation of the kava extract, including what portion of the plant is used, can play a role in this toxicity. There is suggestion that preparations made from the root of the plant are generally safe, whereas other portions of the plant, such as the stems or leaves, might be more toxic [45]. Likewise, there is some speculation that the method of extraction, specifically the solvents used in the process rather than the plant-based compounds themselves, might be the true offending concern [46].

## 6. L-Tryptophan

The effects of the amino acid L-tryptophan on sleep have been investigated since the 1970s with varying support for its role in treating sleep disorders [47]. L-Tryptophan is a precursor to serotonin from its metabolite, 5-HTP. Tryptophan is available in a variety of plant and animal products including eggs, cheese, chocolate, oats, fish, poultry, spirulina, sesame, and sunflower seeds, among many others. Tryptophan is available as an over-the-counter dietary supplement; it is also available in prescription form in Europe, typically as an antidepressant.

Nocturnal administration has been shown to increase concentrations of both serotonin and melatonin; thus its putative mechanism of action may be through either melatonin enhancement or serotonergic effects. Significantly reduced sleep latency and increased subjective ratings of sleepiness in healthy adults during the day have been reported in a number of uncontrolled and controlled studies [48].

Varying doses have been shown to significantly reduce sleep latency and increase subjective ratings of sleepiness in subjects with insomnia [47]. Interestingly, in an experimental tryptophan depletion study in 15 insomnia subjects, tryptophan depletion had a negative impact on sleep continuity, increasing stage 1 sleep and decreasing stage 2 sleep [49]. A systematic review of sixty-four randomized controlled trials exploring the use of alternative modalities to treat insomnia found mixed evidence for L-tryptophan, although this was based on only three studies [50]. In a four-arm RCT (*n* = 96), Hartmann et al. compared one-week administration of tryptophan, flurazepam, secobarbitol, and placebo [51]. Only flurazepam was associated with subjective sleep improvements relative to placebo. Tryptophan did not improve sleep during the 7-day treatment phase, but in the week following treatment discontinuation, sleep latency did improve relative to placebo. In a blinded crossover trial of high (2 g) versus low/placebo dose (0.04 g) L-tryptophan (*n* = 39), Demisch et al. reported a significant difference between full L-TRP dose and the placebo dose, but only if the placebo was given first. There was no group difference when the full dose was received first, suggesting a significant placebo effect [52]. Finally, a three-arm RCT (*n* = 49) compared one-week administration of a food sourced tryptophan (butternut squash seed) to pharmaceutical grade tryptophan and a carbohydrate alone (placebo), all prepared in food bars. In this study, both forms of L-tryptophan resulted in significant improvement on sleep diary measures of insomnia, but similar improvements were noted in the placebo condition such that no significant group × time interactions were observed [53]. The largest improvement was in total sleep time with a 19- minute increase in the squash seed condition and a 42-minute increase in the tryptophan supplement condition, although there was also a 17-minute increase in the placebo condition. In addition, some of the gains were maintained at one-week follow-up. At this time point, placebo was associated with a further increase of additional 24 minutes of total sleep time. Taken together, these trials do not provide convincing support for the efficacy of L-tryptophan for insomnia.

With respect to safety, the sale of tryptophan was banned in the USA from 1991 to 2001 following a large tryptophan-related outbreak of eosinophilia-myalgia syndrome (EMS) leading to 37 deaths. Sales resumed in 2001, but cautions related to worsening of liver and kidney disease remain due to the link to EMS [54, 55]. It is also listed as “likely unsafe” for pregnant or breastfeeding women. On its own, tryptophan is generally safe with mild side effects that include gastrointestinal side effects as well as headache, lightheadedness, drowsiness, dry mouth, visual blurring, muscle weakness, and sexual problems. In addition, there may be major interaction with serotonergic agents such as many antidepressants, leading to serious side effects such as serotonin syndrome. It might act synergistically with sedative-hypnotic medications to cause oversedation; caution should be used when considering with central nervous system depressants as to avoid excessive sleepiness and sedation.

In sum, there is evidence supporting a sleep moderating effect of L-tryptophan. Some safety concerns remain from the EMS outbreak and from the possible severe interaction effects (especially with serotonergic agents), but L-tryptophan is generally well-tolerated with limited side effects. Controlled trials in samples of insomnia patients, however, are quite limited and their combined findings are mixed at best.

## 7. Marijuana

In recent years, there has been significant interest in medical marijuana as a treatment for a variety of medical conditions, especially for, but not exclusive to, palliation. Cannabinoids are thought to exert their effects primarily through a variety of G-couples cannabinoid receptors in the central nervous system and in peripheral tissues. The marijuana plant can contain over 60 cannabinoids, some of which appear to be more bioactive than others [56].

Leaving aside the recreational history of marijuana, the plant has generated significant interest over millennia for its purported medicinal properties. In the United States, the cultivation of the plant was referenced as early as the 17th century. In general, there was a permissive attitude towards the drug, both recreationally and medicinally, until the early 20th century. Marijuana was included in efforts to control more socially dangerous drugs, such as opiates and cocaine throughout the 1900s. It came under increasing regulation, either by taxation or direct restriction, until the 1950s, when both the Boggs Act and the Marijuana Control Act mandated sentences for drug offenders.

Both before and during this time, marijuana has been described for relief of a variety of conditions, including pain, spasticity, emesis, and anorexia. The medicinal use of marijuana has included synthetic cannabinoids in tablet form, as well as the delivery of more “natural” forms of the drug such as smoking or alimentary ingestion. The FDA released a policy statement in 2006 that there was no sound medical evidence supporting the use of marijuana for medical purposes; since that time, 10 states have approved medical marijuana bills into law, and the controversy shows no signs of abating [57].

With this background, there is interest in considering this plant-based drug for the management of sleep disorders. It bears noting that there are significant legal and quality control hurdles in conducting medical research on cannabis [58]. With these limitations, it is important to highlight that the absence of evidence does not necessarily imply evidence of absence.

The literature is sparse with observational studies regarding the effects of cannabinoids on sleep. Many of these reports were published over 40 years ago and are limited by small sample size. Regarding sleep architecture, the evidence about cannabinoid's impact is conflicting. The reports varied in regard to dosage and chronicity of THC administration, leading to a great deal of methodological inconsistency. In general, the case series are consistent in that acute THC administration reduced REM sleep in study subjects [59, 60], although at least one report was not supportive of this finding [61]. There was no agreement as to THC's impact on slow wave sleep, with some studies suggesting increase in this stage and others suggesting decrement or no change [60, 62, 63]. There was no described trend for metrics of insomnia, such as number of awakenings or sleep onset latency (SOL). A few of the papers did describe increased sleep onset latency or wake after sleep onset in the withdrawal state [60, 62, 64]. These observations provide little insight into the mechanisms of sleep regulation of THC. It also bears noting that change in sleep architecture, particularly in regard to total percentages of sleep stages, does not necessarily confer therapeutic advantage. For instance, most commonly prescribed antidepressant medications suppress REM sleep, but this has no known direct detrimental or salutatory sleep effect on the individual patient. Similarly, changes in sleep architecture mediated by THC do not necessarily imply a therapeutic effect.

There is no published evidence that demonstrates benefit of marijuana in the management of primary sleep disorders. Specifically, PubMed searches for cannabis and sleep, cannabis and insomnia, and cannabis and sleepiness did not produce any studies that have explored the impact of cannabinoids on disordered sleep. Likewise, there are no published data that demonstrate an impact of cannabis in treatment for posttraumatic stress disorder, a complex condition with sleep disturbances as an essential feature. Cannabis has been demonstrated in several clinical trials to improve chronic pain in a variety of patient populations [65]. Many of these trials did not include a sleep outcome and any benefit to cannabis on sleep, even in patients with chronic pain, is still speculative.

The list of potential side effects of marijuana is long, politically charged, and beyond the scope of this review. Like any drug, marijuana, with multiple bioactive agents, could certainly be harmful depending on a number of factors. There is evidence for psychological dependency. The inhalation of marijuana smoke has also been implicated in periodontal and pulmonary diseases [66, 67]. These potential drawbacks do need to be considered for a general patient population.

At this time, there is insufficient evidence to prove definitively the impact of THC on sleep architecture or to support the use of marijuana in the management of sleep disorders, but the topic has been subject to almost no scientific scrutiny. Future research should consider these questions, as well as indications for the drug, potential side effects, and the still significant legal hurdles in many states.

## 8. Valerian

Valerian is a perennial herb that has long been held to have sedative properties. The Greek physician Galen recommended valerian for insomnia. The most common species from which valerian is derived is* Valeriana officinalis*. Valerian is extracted from the dried root and rhizomes of this plant by soaking in one of several solutions (water, ethanol and water, or methanol and water) and then either centrifuging or drying the mixture to concentrate the primary active plant compounds including valerenic acids and amino acids [68].

An efficacious dose is considered to be one which has the equivalent of 2 to 3 grams of dried root material. Although commercially available preparations of valerian are most common in tablets ranging in dose from 300 to 600 mg/day and to a lesser extent in 2–4 mL tinctures [69], it is impossible to determine their valerian equivalent when extraction ratios are not provided. One pharmacokinetic study in healthy subjects taking a 600 mg dose of valerian extract, with an undisclosed extraction ratio, observed peak concentrations at 30–120 minutes with a mean elimination half-life of 1.1 hours and its marker, valerenic acid, was observed in serum for up to 5 hours [70].

The precise mechanism of action for valerian is unknown, as is what compound or combination of compounds is responsible for any sedating effect. The main putative pathway is thought to occur through inhibition of sympathetic activity by action upon the neurotransmitter gamma-aminobutyric acid (GABA), although it may also be via binding with adenosine (A1) and/or serotonin (5-HT-5a) receptors [71, 72]. Importantly, like with kava kava, the solution used in the extraction method (the water to alcohol ratio) can dramatically impact the concentration of constituent compounds extracted [73]. Moreover, since valerian is not FDA regulated, the content and concentrations can vary from the listed amount on labels in the United States [74]. Under the auspices of the European Pharmacopoeia, preparation/dosage control in Europe is stricter. In addition, valerian can be prepared in combination with other herbs such as hops (*Humulus lupulus*) or lemon-balm (*Melissa officinalis*), further complicating the assessment of valerian's effect on sleep.

With respect to safety, valerian is on the FDA's GRAS (generally recognized as safe) list and is approved for use as a food. The main secondary effects of valerian include gastrointestinal effects such as diarrhea, abdominal pain, nausea, and pyrosis and central nervous system effects such as headache, nervousness, and drowsiness, although these effects are generally mild and self-limited. With the exception of diarrhea, none have been elevated when compared to placebo in clinical trials [75, 76]. There are no notable interactions between valerian and pharmaceuticals, but caution might suggest avoiding it in combination with benzodiazepines.

The empirical evidence for valerian on sleep remains inconclusive due to contradictory findings. Several studies have found no effect of a single dose of valerian [77–79]. A systematic review of 29 controlled and eight open label trials concluded that, despite some positive studies, the majority of findings were negative and, therefore, that there is no overall effect of valerian, regardless of preparation type or whether or not it was combined with other nutraceuticals [75]. A subsequent review of sixteen studies exploring the treatment of insomnia using valerian with or without hops concluded that twelve studies showed some sleep benefit of valerian [80]. This review included 3 uncontrolled trials. Furthermore, there were significant methodological differences among the thirteen RCTs. Among the RCTs, positive effects of valerian were observed in 1 of 10 assessing total sleep time; 5 of 11 assessing sleep latency; 3 of 6 assessing slow wave sleep; 2 of 10 assessing nocturnal awakenings; and 4 of 13 assessing sleep quality.

A meta-analysis found a statistically greater effect of valerian on sleep quality compared to placebo, but it found that there were no other differences [81]. A subsequent meta-analysis of 18 RCTs, which included three newer randomized trials, found a difference in effect favoring valerian when a sleep quality variable was dichotomized but no difference in sleep quality measured by visual analog scale and no difference in sleep latency [76]. The authors further conclude the most recent clinical trials were of high methodological quality and sufficient sample size, but their combined results were not conclusive.

In the largest trial included in the meta-analysis, Oxman and colleagues (2007) conducted a multicentre trial comparing the equivalent of approximately 3.6 grams of valerian from commercially available valerian tablets to placebo taken for two weeks in 405 adult participants with >1-month insomnia and a score > 5 on the Pittsburgh Sleep Quality Index (PSQI) [82]. There were very few significant differences between groups on the primary and secondary sleep outcomes; the authors conclude that valerian may have a small effect on sleep quality but is unlikely to reduce the time it takes to fall asleep or increase daytime energy level.

Two high quality studies have been published since this large meta-analysis exploring valerian's impact on sleep. In one, 100 postmenopausal women aged 50–60 who had insomnia were randomly assigned to receive twice a day for four weeks 530 mg of a valerian root extract or 50 mg starch as placebo in capsules (although the extraction ratio was not provided, so the valerian dose equivalent is unknown). Nonetheless, in this study, valerian was associated with a greater reduction in PSQI scores over the treatment period (from 9.8 to 6.0) than placebo (from 11.1 to 9.4) and a greater proportion of participants achieving a 5-point reduction in their PSQI score (30% versus 4%) [83]. In another, 91 patients diagnosed with primary insomnia, perceived total sleep time of <6 hours, and an Insomnia Severity Index score > 7 were randomized to receive a 10 mg zolpidem tablet or one tablet containing 300 mg of a valerian extract (standardized to 0.8% valerenic acid), hops, and passion flower (*Passiflora incarnata)* extracts at bedtime for two weeks [84]. Thirty-nine subjects in each group completed the study, which found significant improvements on sleep diary-measured sleep latency, total sleep time, and nocturnal awakenings as well as significant improvements on the ISI in each group. There were no time by group differences on any of these measures, suggesting equivalency between zolpidem and this particular formulation of valerian, hops, and passion flower.

In sum, the evidence to support the use of valerian as a sleep aid for patients with insomnia remains rather mixed, with more negative than positive studies overall. A few high quality studies now report modest benefits of valerian for insomnia patients. The safety of valerian is now well-established.

## 9. Summary

From even this basic review of a few of the nutraceutical supplements available for sleep disorders, it is clear that there is a spectrum of evidence to support their use. With few exceptions, a general theme with these agents is that there are historical and anecdotal reports of sleep benefit, but the science falls significantly short of class IA evidence. In fact, some of the agents have essentially no published data at all to support their use, even as case reports. The National Institutes of Health has National Center for Complementary and Integrative Health (NCCIH), with a 2015 budget of 124.1 million dollars, about two one-hundredths that of the National Cancer Institute [85]. For those who have an interest in these plant-based products for use in sleep disorders, the hope is that some of this modest budget along with other support will be granted for these types of research.
